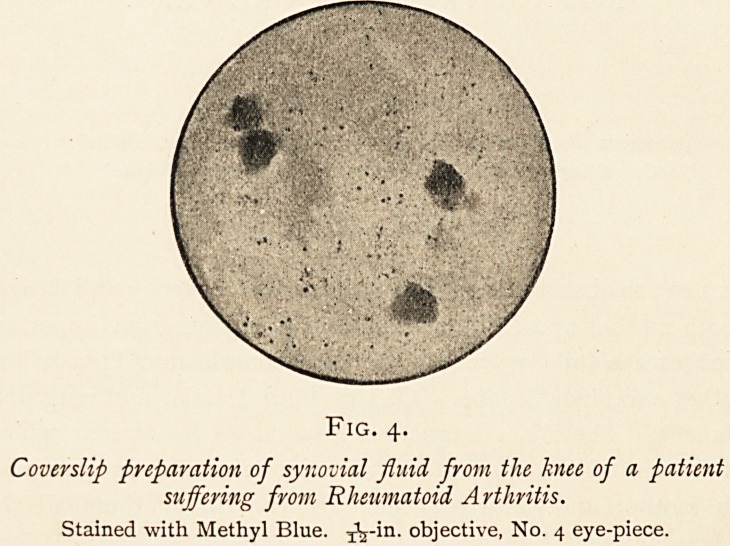# The Clinical Significance of the Human Hand

**Published:** 1896-06

**Authors:** Arthur S. Wohlmann


The Bristol

Medico-Chirurgical Journal.

JUNE, 1896.

### THE CLINICAL SIGNIFICANCE OF THE HUMAN HAND.

BY

Arthur S. Wohlmann, M.D., B.S. Lond.

The hand is so important a part of the human anatomy, it has played so large a part in the evolution of the human race, and has so largely contributed to the eventual triumph of man over his brute competitors, that it must always have an especial interest for us, even in the study of our always interesting bodies.

When first a patient comes before us—even before we have felt his pulse or wisely shaken our heads over his extended tongue—we have from force of habit learned much of him by a hasty critical analysis. Almost unconsciously we have noted his general vitality, compared his height and weight—his slight stoop, perhaps, or the thickness of his great omentum.

Unconsciously we have studied his face, and have gleaned therefrom something of his character, of his present condition, often of his past history. In the note-book of the mind we have already jotted down shorthand memoranda as to his cyanosed lips, the mottled venules of his cheeks, or his yellowed conjunctivæ: notes which, used aright, may serve as finger-posts to direct our enquiries, but which also may lead us astray if too hastily we jump to conclusions. The face then we examine naturally, and almost without consciously turning our attention upon it, and the importance of so doing is so obvious that further comment on the point is superfluous.

But the face is not the only portion of the anatomy deserving of scrutiny; and what I wish to point out is, that a routine examination of the hands in all cases will well repay the slight extra labour involved, and will, after a time, be made almost as quickly and unconsciously as the glance at the face.

The basis of all the remarks to follow is the axiom "that in all our tissues there is no such thing as a *perfect* elasticity," and that consequently every muscular action, nay every passing thought, leaves some mark behind in our body generally, some slight track in the labyrinth of our brain-paths. The mark may not be appreciable—if not repeated; it may be apparently effaced by the myriad contrary impressions that follow, but even so, in some infinitesimal degree it must modify the subsequent impressions; but suppose it be repeated, then in course of time we get an imprint manifest and unmistakable. The facial expression is a case in point. Every passing wave of emotion finds its haunting shadow in the contraction of a few fibres of the ever-busy mesh of facial muscles. Whether the emotion precedes the muscle-contraction, or the muscle-contraction precedes and causes the feeling of emotion, is a point over which psychologists still fight. In any case, oft repeated thoughts, emotions, and contractions "set" the face in certain well-recognised lines of expression. And we of modern days and Anglo-Saxon impassivity, who pride ourselves that our passing thoughts and emotions are hidden by a mask of reserve from the vulgar crowd, do not we, by this very reserve, by this inhibition of muscular action, call forth other muscular actions, and so imprint on our faces some sort of expression after all?

In this respect the hand closely resembles the face. In both cases we find a large number of highly specialised small muscles, in both cases these are in close connection with special sense organs; for what, after all, are the finger-tips but special organs of touch? The remarks then that applied to the face apply, though perhaps with less force, to the hands. In the one case, by long custom we have learned to read the open page with some degree of ease and certainty; in the other, we are yet as little children stumbling over the alphabet. Perhaps after all, deep hidden in the muddy depths of charlatanism, there may be some pearl of truth in the study of palmistry! Indeed, these minuter scrutinies have already been largely utilised in criminal anthropology. The remarkable persistence of the epidermal lines of the finger-tips, as pointed out by Galton, is made of every-day use in the identification of criminals; while Lombroso has written eloquently of the criminal expression shown by the hands and other parts, just as by the face, though I think that most unbiassed observers will allow that his enthusiasm outruns his discretion.

In the by-path of the foregoing psychological considerations I meant to point the moral that if mental conditions can so affect the configuration of the hands, how much more will actual organic diseases!

Naturally some diseases will affect them more, some less. The latter, for our purposes, are beyond the scope of a short paper like this. All that I propose is, to draw attention to those features that are writ large, so to speak, that are easily to be seen at a glance, and that above all are useful aids in diagnosis.

Now, I do not propose to give a catalogue of all the diseases which affect either solely or largely the hands, but rather to draw attention to their condition in two great groups of diseases: one, the obscure group of which we may take acromegaly as a type; the other, the far more important and numerous group comprised under the heading "Rheumatic," using the word in its widest sense.

There is a general rule, first definitely formulated I believe by Arbuthnot Lane, which applies to the bones of the hands as it does to the skeleton generally. "Deformity is first an exaggerration, and then a fixation, of the normal physiological position of rest." There are, of course, exceptions to this as to every other rule, but in the main it will be found to hold true, and to afford a simple and ready explanation of many apparently fortuitous deformities, and a rule that will enable us to forecast the direction of those impending. Take for instance curvature of the spine: see how first the weakened column adopts habitually the easy curves of rest, how these curves then become exaggerated, and then gradually fixed and permanent. Take the knee: the easy, restful, relaxed position is semiflexion; the natural end of a severely affected knee is fixation in a *more* than semiflexed position. In the wrist the restful position is midway between flexion and extension; and in a straight position the wrist is generally ankylosed. The ulnar deflection of the hand in chronic rheumatism is well known. Is not the normal position one of slight ulnar deviation? Of course, Nature does not bind herself down to always work according to one simple rule; a thousand other influences are at work complicating the main issue, but all working to a definite conclusion, and all equally capable of scientific analysis. According as we look more closely into the matter, so does the element of chance as a causative factor diminish. Take the worn bones of a man who has long and incessantly toiled at the same trade: we soon come to recognise certain deformities peculiar to certain trades—ankyloses and false joints, erosions and bosses of bone, which we can readily see must necessarily have arisen as a consequence of the habitual assumption of the same position; in fact we can often by the deformities and so-called abnormalities of a skeleton determine the occupation of its erstwhile owner, just as a comparative anatomist may perform the sensational feat of building up an animal from a single bone. In this connection the well known "housemaid's knee," "miner's elbow," and "carpenter's hollowed sternum" will at once flash across the mind.

Arbuthnot Lane has shown how in many, if not in most cases, the changes set down to osteo-arthritis are really due to a traumatic arthritis whose mechanism can be readily worked out on physical laws, and its relation shown to the patient's occupation or to a definite traumatism.

I will now consider the condition of the hands in the various rheumatic diseases, using the term "rheumatic" in its widest sense. The diagnosis between gout, rheumatism, and rheumatoid arthritis is often one of extreme difficulty; and it is here

FIG. 1.
*Gout.*

FIG. 2.

*Rheumatism.*

FIG. 3.
*Rheumatoid Arthritis.*

that the observation of the hands is of the very greatest utility. Fig. 1 is from the cast of a gouty hand which I took from a middle-aged woman some two years ago, and painted from life. It is rather a rare specimen. Actual concretions of urate of soda, even in great masses, are common enough in men, though, with the increased attention paid to personal health and comfort, I think they are tending to become less so year by year; but in women, although men have no monopoly of gout, such concretions are very uncommon, and in looking back through my notes of several hundred hospital cases I find mention of only one other. Of course, with such a hand as that before one, the diagnosis is almost insolently self-assertive; but in less pronounced cases we get a couple of bony-looking nodes at the base of the terminal phalanges, some irregular hyperextension and side-twisting of the fingers, or some thickening and striation of the nails due to impaired metabolism. In this latter connection a patient of mine assures me that with every prolonged attack of gout his finger-nails cease growing, to start growth again after the last effects of the attack have passed off.

Fig. 2 is from one of the casts of some hands kindly lent me by Dr. Preston King. It is fairly typical of the group of diseases which, for want of a better name, we lump together as "chronic rheumatism." Worthy of note in this specimen are the marked ulnar deviation of the fingers and the hyperextended phalanges. In this group the hands are generally symmetrically affected, a most important distinction from the hands of gout, as pointed out in a recent number of the *British Medical Journal* by Dr. Ernest Reynolds. The bosses about the hands are hard and bony, osteo-arthritic in fact, and quite different in their nature from the soft doughy swellings in the delicate cast (Fig. 3) which I took from a young woman with rheumatoid arthritis. In the other cases, except where actual tophaceous deposits were visible, we had to rely rather on probabilities than certainties for diagnosis. In the case represented by Fig. 3, there is no room for probability, it is absolute certainty. There is spindle-swelling of the middle joints of the fingers—those spindle joints would be soft to the touch, semi-elastic, and probably hot, with a slight bluish blush in the skin over them; the thickened wrist—likewise soft—and the wasted interossei will be noticed. The palm would be cold and wet, the fingers leaden-hued and asphyxiated; perhaps there would be pigment-spots over the dorsum. Lastly, both hands would exhibit the most marked symmetry in their lesions. If left to Nature, the ultimate end of such a hand would approximate very closely in its pathological aspects to the final conditions of the hand affected by chronic rheumatism; both would end in an osteo-arthritic condition characterised by lipping and bossing of bone, erosion, and great deformity. In this stage, which is comparatively painless, the diseases have spent their strength, and both find a common grave in osteo-arthritis.

But though the end of both diseases is pathologically the same, *clinically* they are usually readily to be distinguished. The hand affected by chronic rheumatism may be distorted into all sorts of curious shapes, partly to be explained by the laws already given, partly due to pressure of bony outgrowths or to changes in ligaments and atrophy of supporting muscles. The hand erstwhile in the throes of rheumatoid arthritis may exhibit much the same lesions, due to the same forces at work, but above all, and masking all, is generally to be seen the original deformity—the characteristic swellings, once soft and impressionable, now hard, calcareous, and fixed. Tubercular dactylitis may at first sight closely simulate rheumatoid disease in children; but the spindle-swelling is caused by bone expansion, and is accompanied by suppuration, in both of which points it differs essentially from the latter disease.

The other group, the rarer diseases, I shall dismiss in a few words.

1. Paget's disease (osteitis deformans). The cranium and long bones are the parts usually affected; and though the hands sometimes show changes, these are hardly diagnostic and are very rare.

2. Acromegaly (of Marie). The large size of the hands is due to the excessive development of *all* the tissues. There is no appreciable increase of length, only of width and thickness, earning for the hands the epithets of "battledore" and "spade-like." The wrists are about normal. The nails are somewhat flattened, small, and longitudinally striated.

3. Hypertrophic pulmonary osteo-arthropathy. The carpo-metacarpus, the hand proper, is about normal in size; but the fingers are enormously enlarged, the bulbous terminal phalanges being especially prominent. The nails are curved and striated, reminding one of the beak of a parrot. There is great enlargement of the wrists.

4. Myxœdematous hands may at first sight be mistaken for either 2 or 3; but it will be seen that the skin of the other parts of the body is involved, and it is adherent to the deeper structures.

5. Vaso-motor paralysis of the extremities. A few rare cases have been set down to this cause, and may somewhat resemble the foregoing: the presence of subcutaneous hemorrhages may clear up the diagnosis.

6. The hands may exhibit marked changes in rickets, which in rare cases may simulate some of the former diseases; but an examination of the rest of the body will generally readily afford the data for a diagnosis.

7. In leontiasis ossea (Virchow) the hands may be affected; but for diagnostic purposes the changes are unimportant.

8. Nodosities of the fingers have been noted in connection with dilatation of the stomach.

I do not pretend to have exhaustively treated even one aspect of the subject, and I have left untouched the clubbed fingers of emphysema, abnormal and extra digitation, Dupuytren's contraction, Raynaud's disease in connection with rheumatism, and all the thousand and one things that crowd to one's mind when it is allowed to rest on a subject. I hope that this paper, which is rather of the nature of a sketch than a finished study, will tend to stimulate and systematise observation of these interesting cases.

---

### NOTE ON THE BACILLUS OF RHEUMATOID ARTHRITIS.

As the foregoing paper bears so largely on the question of the differential diagnosis of Rheumatoid Arthritis and the other "rheumatic diseases," it was suggested to me that I should give a short *resumé* of the subject of the Bacillus of Rheumatoid Arthritis, concerning which an article by Dr. Bannatyne, Dr. Blaxall, and myself appeared in the *Lancet* of April 25th. Theories of causation have clustered thick about the name Rheumatoid Arthritis, but hitherto most authorities have been inclined to look upon it as due to some obscure nervous lesion. It always seemed to me, however, looking at the disease from the clinical standpoint, that it more closely resembled some of the diseases of proved microbic origin such as tubercle, and some two years ago, after much fruitless search, Dr. Bannatyne and I were rewarded by finding a distinctive microbe in the synovia of affected joints. Hitherto, owing to its small size and peculiar staining properties, this had escaped our observation; but on looking back again through our old specimens we frequently came across it. Briefly, our method of procedure was as follows: A drop of fluid, aspirated with aseptic precautions from a distended joint, was stained in thin film on a coverslip, various nutrient media were inoculated at the bedside, and, if sufficient fluid had been obtained, a reserve was set aside in sealed glass tubes for further examination. At first we used fairly strong solutions of fuchsin, floating the coverslip on the warm stain for two or three minutes. By this method the organisms were rapidly and deeply stained, but so also unfortunately was the synovial film; and although thorough washing in water or in very dilute acetic acid removed much of the ground-stain, yet the bacilli themselves were generally decolourised almost as rapidly, so that we were rarely successful in getting satisfactory specimens. Substituting weak solutions of methylene blue for fuchsin, and staining for longer periods, up to five days, we got better results. The organisms were more clearly defined, but seemed distinctly smaller than when stained with fuchsin. So far, however, we have been quite unable to find a distinctive and selective stain. Gram's method entirely fails; and though many stains readily colour the microbe, they are all as readily washed out. After working at the subject for nearly a year, and finding that, although we distinguished the microbe in nearly every rheumatoid joint examined, we were yet unable to secure satisfactory cultures, we came to the conclusion that we could go no further with our somewhat primitive apparatus, and placed the matter in the hands of Dr. Blaxall, of the British Institute of Preventive Medicine, for further investigation. He confirmed our results, obtained cultures, though only after much labour, and observed division of the microbe in the hanging drop; he also detected the bacillus in several specimens of blood that we sent him. A full report of his work will be found in the paper referred to; and I will only give here a short summary of the appearance and life-history of the organism. It is a short bacillus, averaging 2 $\mu$ in length by .6 $\mu$ in breadth, though both these dimensions are liable to considerable variations dependent upon the maturity of the organism and the staining reagent employed. It is dumb-bell in shape, the connecting bar being exceedingly difficult to stain, and generally remaining invisible, the two stained poles giving the appearance of a diplococcus. (See Fig. 4.) It is non-motile, but exhibits marked oscillatory movements. In the hanging drop

Dr. Blaxall has observed the process of division, and I cannot do better than quote his own description: "The intervening portion lengthens out, the ends appearing to pull against one another energetically, the whole organism oscillating the while

FIG. 4.

*Coverslip preparation of synovial fluid from the knee of a patient suffering from Rheumatoid Arthritis.*

Stained with Methyl Blue. $\frac{1}{12}$-in. objective, No. 4 eye-piece.

uneasily. The middle part lengthens out more and more, so that the organism appears to be almost twice its ordinary length, then suddenly the link snaps, and the freed ends fly off in contrary directions and are lost amid their fellows."

*Cultivation.*—In flasks of perfectly clear peptone beef-broth at blood-heat very minute floating colonies appear on the fourth day, scarcely visible and resembling "gold-dust" when the flask is held up to the light. Tubes of sloping nutrient agar-agar inoculated from the beef-broth show on the fourth day a delicate translucent superficial film, much as if one had breathed on a polished surface. A similar growth will occur on blood-serum, but is more difficult to discern.

The following conclusions have been arrived at:

(1) This organism has been found in the synovial fluid in 24 out of 25 cases examined.

(2) It has also been seen in the blood in a few instances.

(3) It is constant in appearance, and exhibits marked peculiarities of growth.

(4) It has not been found in synovitis due to other causes, though organisms morphologically somewhat similar have undoubtedly been noted.

For these reasons, although the crucial test of animal inoculation has not yet been fully worked out, there would seem little reason to doubt that this bacillus is the true cause of rheumatoid arthritis.

## Figures and Tables

**Fig. 1. Fig. 2. f1:**
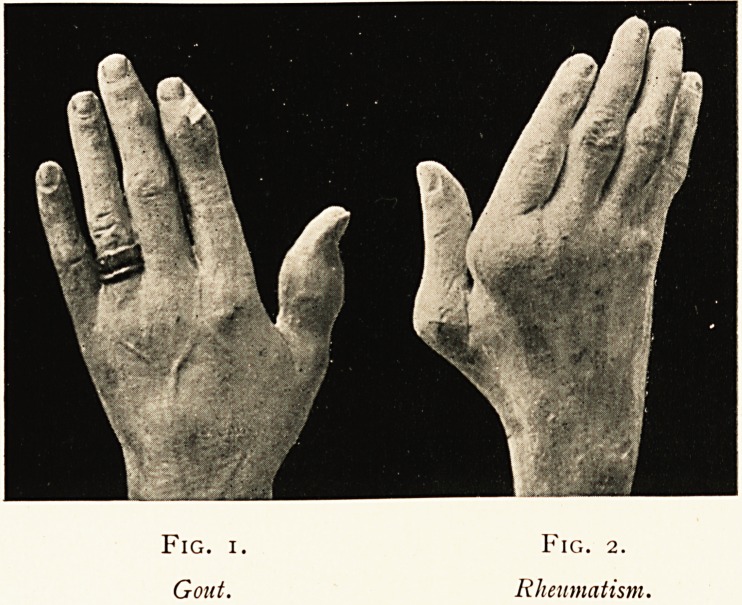


**Fig. 3. f2:**
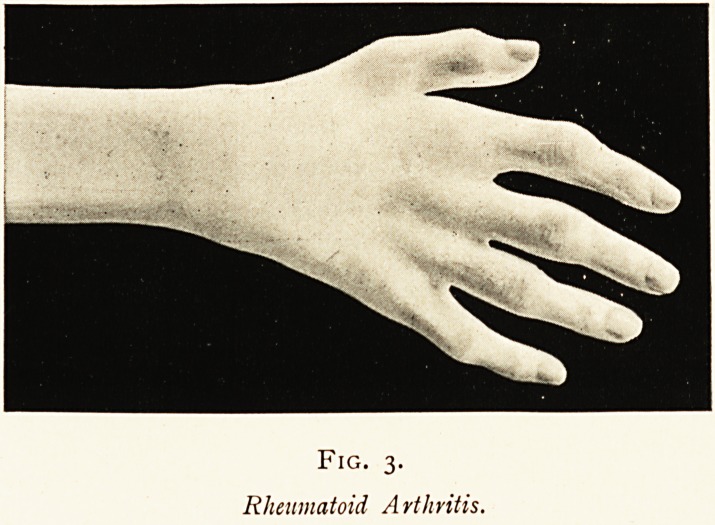


**Fig. 4. f3:**